# Interleukin‐1β Stimulates Matrix Metalloproteinase 10 Secretion: A Possible Mechanism in Trophoblast‐Dependent Spiral Artery Remodeling

**DOI:** 10.1096/fj.202402329RR

**Published:** 2025-05-06

**Authors:** Holly Tinsley, Zoe Tryfonos, Miriam Aziz, Nora Lagzouli, Charlotte Longhurst, Alexander Frick, Sandra Ashton, Judith E. Cartwright, Guy S. Whitley

**Affiliations:** ^1^ Centre for Vascular Biology, Cardiovascular and Genomics Research Institute St George's, University of London London UK; ^2^ Royal Devon University Trust Exeter UK; ^3^ Fetal Medicine Unit St George's University Hospitals NHS Foundation Trust London UK

**Keywords:** endothelial, MMP‐10 (MMP10), spiral artery, stromelysin‐2, trophoblast

## Abstract

Maternal uterine spiral arteries (SpA) undergo significant structural changes in early pregnancy, resulting in increased blood flow to the developing fetus. Endothelial cells (EC) and vascular smooth muscle cells (VSMC) are lost from the SpA wall and are replaced by trophoblasts. We have previously shown that matrix metalloproteinase 10 (MMP‐10) and Heparin binding‐EGF like growth factor (HB‐EGF) gene expression is increased in a 3D EC/VSMC co‐culture system in response to trophoblast secreted factors. This study investigated trophoblast mediated MMP‐10 and HB‐EGF expression and determined if there was a relationship between the secretion of MMP‐10 and the release of soluble HB‐EGF (sHB‐EGF) from EC. MMP‐10 was widely expressed in first trimester decidual tissue including trophoblast, and EC, but not VSMC. MMP‐10 expression was significantly lower in decidual tissue from pregnancies at increased risk of developing pre‐eclampsia compared to low‐risk pregnancies. In vitro, SGHEC‐7 cells, a human EC line, but not SGHVMC‐9, a human VSMC cell line, secreted MMP‐10 in response to trophoblast conditioned medium (TCM). TCM contains several growth factors and cytokines, but only interleukin‐1β (IL1β) significantly stimulated MMP‐10 secretion by SGHEC‐7 cells. Interleukin‐1 receptor antagonist (IL‐1Ra) significantly inhibited TCM‐induced MMP‐10 secretion. Interrogation of intracellular pathways established the involvement of MEK and JNK in TCM and IL‐1β stimulated MMP‐10 secretion. Although IL‐1β also significantly increased sHB‐EGF, inhibition of MMP‐10 activity using a broad spectrum MMP inhibitor had no effect on sHB‐EGF. Western blot analysis indicated that MMP‐10 secreted by EC in response to IL‐1β stimulation was the enzymatically inactive pro form.

## Graphical abstract

Summary of the pathway intermediates involved in the secretion of MMP10 by endothelial cells in response to stimulation with trophoblast conditioned medium and interleukin‐1β.

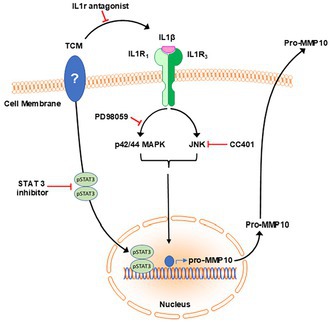

**Abbreviations**
BSA: bovine serum albumin
DAB: 3,3′‐diaminobenzidine
EC: endothelial cell
FCS: fetal calf serum
HB‐EGF: heparin‐binding EGF‐like growth factor
HIF: hypoxia inducible factor
IL1‐β: interleukin 1 β
IL‐Ra: interleukin receptor antagonist
MMP: matrix metalloproteinase
NHS: National Health Service
NNGH: N‐Isobutyl‐N‐(4‐methoxyphenylsulfonyl)glycyl hydroxamic acid
PBS: phosphate buffered saline
PBST: phosphate buffered saline with tween
sHB‐EGF: soluble heparin‐binding EGF‐like growth factor
SpA: spiral artery
TBS: Tris buffered saline
TBST: Tris buffered saline with tween
VSMC: vascular smooth muscle cell

## 1 Introduction

To meet the increasing demands of the growing fetus for nutrients and respiratory gases, blood flow to the placenta must increase [1]. This is achieved through significant changes to the structure and cellular nature of the maternal uterine spiral arteries (SpA) that supply blood to the placenta. These changes are characterized by the loss of the extracellular matrix (ECM) from the basement membrane, vascular media, and adventitia [2]. These changes are initiated by maternal macrophages and uterine natural killer cells that accumulate around the vessels. However, complete remodeling requires cells derived from the placenta, termed extravillous trophoblasts (EVT) [3, 4]. Failure of EVT to invade and remodel the vessel wall adequately results in poor placental perfusion, which is characteristic of several common obstetric disorders, including early pregnancy loss, pre‐eclampsia (PE), and fetal growth restriction (FGR) [5].

EVT migrate from the anchoring villi as two distinct populations. They invade either by an interstitial route through the decidua or by an endovascular route via the distal ends of the SpA. Endovascular EVT interact initially with the EC of the vessel, whereas the interstitial EVT first encounter the VSMC layer. The cellular changes that take place during the remodeling process include trophoblast‐dependent apoptotic cell loss [6, 7], dedifferentiation of VSMC [8], changes in adhesion molecule expression [9] and disruption of cellular interactions [10]. However, EC and VSMC are not passive observers in the remodeling process and respond to trophoblasts with alterations in protease production [11], cell motility [12] changes in adhesion [13] and cytokine production [14]. In a non‐pregnant vessel, the ECM consists of several components including collagen and elastin, which promote stability and provide elasticity [2]. Previous studies have identified the expression of several proteolytic enzymes at the maternal‐fetal interface that may be involved in remodeling the maternal SpA including the matrix metalloproteinases MMP‐2 and MMP‐9 secreted by trophoblasts [15], and MMP‐12 secreted by VSMC [10, 16].

To dissect the specific signaling pathways responsible for mediating the effects of trophoblasts on vessel remodeling, we have previously reported the use of a 3D multi‐cellular spheroidal model where a monolayer of EC forms over a core of VSMC, demonstrating the same phenotypical qualities of blood vessels. This model more closely resembles the vascular architecture exhibited in vivo [17, 18]. Genome‐wide microarray profiling established 101 differentially expressed genes between vascular spheroids cultured with trophoblast‐conditioned and control medium. Gene ontology identified a number of genes associated with vascular development, including matrix metalloproteinase 10 (MMP‐10) and heparin‐binding EGF‐like growth factor (HB‐EGF) [19].

MMP‐10, a stromelysin, has been implicated in several biological processes including the degradation, of collagen types III, IV, and V, and fibronectin, elastin, laminin, as well as gelatins formed from several denatured collagen types [2, 20], proteolytic activation of other MMP family members, MMP‐1, ‐7, ‐8, and ‐9 [21], and the release of biologically active molecules from the cell surface [22]. HB‐EGF can be released from the cell surface and act as a soluble factor or can remain membrane‐bound to exert its effects. At the maternal‐fetal interface, HB‐EGF has been implicated in vascular remodeling and in the stimulation of trophoblast invasion and migration through its effect on MMP expression and secretion [23, 24]. In recent studies, MMP‐10 has been implicated in the release of HB‐EGF from the cell surface [22]. The aim of this study was therefore to investigate the regulation of MMP‐10 expression further and determine whether the release of MMP‐10 from vascular cells could influence the release of sHB‐EGF.

## 2 Materials and Methods

### 2.1 Cell Culture

Experiments used the endothelial line SGHEC‐7, derived from human umbilical vein endothelial cells [25]. These ECs were maintained in Medium 199 supplemented with Earle's modified salts (M199 Sigma‐Aldrich UK Cat# M2154): Roswell Park Memorial Institute 1640 medium (RPMI 1640 Sigma‐Aldrich UK Cat# R8758) in a ratio of 1:1 supplemented with L‐glutamine (2 mmol/L), penicillin (100 IU/mL), streptomycin (100 μg/mL), endothelial cell growth supplement (2.5 μg/mL, Sigma‐Aldrich UK Cat# E0760) and 5% (v/v) fetal calf serum. The human vascular smooth muscle cell line, SGHVSMC‐9, was derived by transfection from human aortic vascular smooth muscle cells [26] and was grown in Hams F10 medium (HAMS F10 BioSera Cat# LM‐H1045/500) supplemented with 10% (v/v) fetal calf serum, penicillin (100 IU/mL) and streptomycin (100 μg/mL).

### 2.2 Trophoblast Conditioned Medium (TCM)

TCM was produced as previously described [19]. SGHPL‐4 cells (RRID:CVCL_0521), a first‐trimester extravillous trophoblast‐derived cell line, were grown in 3‐dimensional culture as previously described [27]. In brief, 4 × 10^6^ cells were added to 25 mL Universal tubes containing 20 mg of gelatin‐coated Cytodex‐3 microcarrier beads (Sigma‐Aldrich Cat#: C3275) and cultured on a spiralex roller at 37°C in phenol‐red‐free RPMI‐1640 (Sigma‐Aldrich Cat#R7509) containing 10% (v/v) FCS and 25 mM HEPES buffer. After 24 h, the medium was removed, and the cells and beads were washed twice with serum‐free culture medium. Medium was then replaced with phenol‐red‐free RPMI‐1640 containing 25 mM HEPES (Corning Cat#25‐060‐CL). Trophoblast conditioned medium (TCM) was collected after 48 h and concentrated using Vivaspin 20 (3000 MWCO PES) columns at 4°C. The concentrated media was then made up to ×1 with phenol‐red‐free RPMI‐1640, and the centrifugation step was repeated. The final concentration of TCM was approximately 20‐fold. The protein concentration of the resulting concentrated TCM was determined by Bradford assay, and aliquots were stored at −20°C prior to use.

### 2.3 Identification of Factors in TCM Using Protein Array

Growth factors and cytokines present in TCM were identified using Proteome Profiler Human Cytokine Array Kit (R&D Systems Cat#ARY005B) and Proteome Profiler Human Angiogenesis Array Kit (R&D Systems Cat#ARY007). The Proteome Profiler and Antibody Arrays were performed as directed by the manufacturer's instructions.

### 2.4 Immunohistochemistry

Paraffin embedded placental tissue sections (5 μm) were cut from and mounted on slides and immersed twice in xylene for 5 min. The slides were then rinsed with 100% ethanol and then re‐hydrated to PBS using a series of ethanol dilutions including 100%, 90%, 80%, 70%, 50%, and 30% (v/v). Antigen retrieval was achieved following a 40 min incubation with proteinase K solution (20 μg/mL, Invitrogen). Slides were washed once with PBS and blocked with PBS containing 10% (v/v) goat serum (GS; Vector Labs, Peterborough, UK) for 20 min at room temperature.

### 2.5 Fluorescent Immunohistochemistry

Placental sections were prepared as previously described, with the exception that antigen retrieval was achieved using 10 mM Tris–HCl at pH 10. Smooth muscle actin antibody (Agilent Cat# M0851, RRID:AB_2223500) was used at a concentration of 0.071 μg/mL, and CD31 antibody (Abcam Cat# ab28364, RRID:AB_726362) was used at a concentration of 0.26 μg/mL, and MMP‐10 antibody (Enzo Life Sciences Cat#BML‐SA434, RRID:AB_10539415) at 4 μg/mL. After washing 3 × 5 min with TBS, the slides were incubated for 1 h at RT with secondary antibodies: goat anti‐Rabbit, Alexa Fluor 488 (Thermo Fisher Scientific Cat#A‐11034, RRID:AB_2576217) and goat anti‐Mouse, Alexa Fluor 546 (Thermo Fisher Scientific Cat#A‐11030, RRID:AB_2737024), both at a 1 in 2000 dilution in TBS/1% BSA(w/v)/0.025%(v/v) Triton X100. Slides were then washed 3 × 5 min each with PBS and then mounted with a cover slip. In parallel, consecutive tissue sections were prepared using suitable IgG controls at the same concentration as the CD31(Abcam Cat# ab28364, RRID:AB_726362) and smooth muscle actin antibodies (Agilent Cat# M0851, RRID:AB_2223500). Nikon A1R Confocal Laser Scanning Microscope (RRID:SCR_020317) was used to capture the fluorescent images.

### 2.6 Assessment of the Secretion of MMP‐10 and HB‐EGF

EC and VSMC were grown overnight in 0% FCS phenol‐free RPMI and then stimulated with 0–100 ng/mL TCM in the same medium. TCM also contains MMP‐10; therefore, to prevent this from affecting the subsequent analysis, the medium was removed after 2 h, and the cells were washed extensively with PBS before the medium was replaced with phenol‐free RPMI‐1640 containing 5% (v/v) FCS. Cells were then cultured for up to 72 h before the medium was removed and centrifuged at 14 000 *g* for 10 min at 4°C. The supernatant was removed and stored at −20°C until the MMP‐10 could be determined by ELISA (R&D Systems Cat# DY910). We were able to confirm the manufacturers' data that there was little or no cross‐reactivity when 50 ng/mL recombinant human MMP‐3 (Thermo Fisher Scientific Cat# 420–03‐10UG) was used.

To investigate the intracellular pathways involved in TCM and IL1β stimulated MMP‐10 secretion, experiments were performed with or without pharmacological modulators of common pathways including p42/44‐MAPK, p38‐MAPK, and JNK. Endothelial cells were incubated for 20 min with either PD98059 (CalBiochem, Cat# 513000), SB203580 (CalBiochem, Cat# 559395), CC401 (Biotechne, Cat# 6258), or dimethylsulphoxide (DMSO) as the vehicle control prior to the addition of either TCM (50 μg/mL) or IL‐1β (5 ng/mL). The cells were then stimulated for a further 2 h before being washed twice with PBS. Fresh medium without the stimulus or the inhibitors was then added, and the cells were incubated for a further 24–48 h in phenol‐free RPMI‐1640 containing 5% (v/v) FCS. MMP‐10 secretion into the medium was determined by ELISA. The cells were lysed in RIPA buffer containing a protease inhibitor cocktail of aprotinin (60 μg/mL), phenylmethylsulphonyl fluoride (PMSF; 1 mM) and sodium ortho‐vanadate (1 mM) before sonication for 5 s using an Ultrasonic Processor FB‐120 (Fisher Scientific) set at 60% amplification. The cell lysates were then centrifuged at 14 000 *g* for 10 min at 4°C. Total protein concentrations were determined using Bradford Protein Assay Reagent (Sigma‐Aldrich Cat#B6916‐500ML).

To assess sHB‐EGF, cell media supernatants were concentrated using Sartorius Vivaspin 6 mL 3000 MWCO PES centrifugal concentrator tubes at 4000 *g* for approximately 4 h, or until the concentrated volume reached less than 200 μL. HB‐EGF was detected by ELISA using an HBEGF Duo Set (R&D Systems UK Cat#DY259B). To investigate the possible involvement of MMPs in the release of sHB‐EGF, cells were incubated in the presence and absence of the broad‐spectrum MMP inhibitor, N‐Isobutyl‐N‐(4‐methoxyphenylsulfonyl)glycyl hydroxamic acid (NNGH; Enzo Life Sciences, Cat# BML‐PI115) for 20 min prior to the addition of IL1‐β. Samples were then treated as before.

### 2.7 Doppler Ultrasound Scanning of First Trimester Uterine Arteries

Doppler ultrasound screening of uterine arteries was performed on women undergoing elective surgical termination of pregnancy at St George's University Hospital, NHS Foundation Trust. Inclusion criteria for the study included singleton pregnancy, gestational age 8–14 weeks, normal fetal anatomy and nuchal translucency thickness, and no known maternal medical condition or history of recurrent miscarriage. The gestational age was calculated by crown‐rump length measurement. Doppler ultrasound was performed by a trained sonographer as described previously [28]. Following a study of 10 000 ongoing pregnancies, we have established reference ranges of the resistance indices [29]. In this study, a high‐resistance index (RI) was defined as a pregnancy with bilateral uterine artery notches and a mean RI ≥ 95th centile, while a normal‐RI presents no uterine artery notches and a mean RI < 95th centile. The risk of developing PE would be five times greater in the high‐RI group had the pregnancy continued to term. Experiments used tissue and cells isolated from human placenta obtained following termination of healthy first trimester pregnancies. Ethical approval for this work was covered under IRAS project ID: 285005, REC reference: 20/EM/0246. Involvement in the research study did not alter the clinical care pathway of participants beyond the addition of slightly longer transabdominal ultrasound that was already part of the routine pathway. Clinical data is summarized in Table 1.

**TABLE 1:** Summary of clinical data.

Risk of developing pre‐eclampsia	Maternal age in years	Gestational age in days	Uterine artery RI
Normal risk *N* = 8	28.13 ± 2.24	73.13 ± 3.79	0.76 ± 0.038
Increased risk *N* = 8	27.71 ± 2.66	66.14 ± 3.11	0.9 ± 0.02**

** *p* < 0.01 significant difference between normal pregnancies and those at increased risk of developing pre‐eclampsia.

### 2.8 Western Blot Analysis for Cells and Tissue

After incubation, cells were lysed in up to 200 μL of RIPA buffer containing 100 mM sodium orthovanadate, 17 mg/mL aprotinin, phosSTOP (Roche Cat# 05892970001) and 10 mg/mL phenylmethylsulfonyl fluoride sonicated, and centrifuged at 14 000 *g* for 5 min, and the supernatant collected. Decidual tissue (5–10 mg) was homogenized in 1 mL of RIPA buffer using a MP Biochemicals FastPrep‐24 homogenization (RRID:SCR_018599) tube containing Lysis Matrix D (MP Biochemicals Cat#6913060). Protein concentrations were determined using the Bradford protein assay. Approximately 30–50 μg of protein per well was resolved on a polyacrylamide gel before transfer to an Immobilon‐FL transfer membrane (Millipore, UK). Non‐specific reactivity was then blocked with Tris‐buffered saline (TBS) with 5% (w/v) low‐fat milk powder for 1 h at room temperature. Blots were probed with the following antibodies: MMP10 1:600 (R and D Systems Cat# MAB9101, RRID:AB_2144565) and Tubulin 1:10 000; (Abcam Cat# ab7291, RRID:AB_2241126) in 5% (w/v) low‐fat milk powder in TBS containing 01% (v/v) Tween 20. Protein loading and transfer efficiency were followed using tubulin.

The blots were then washed in TBST and incubated with goat anti‐mouse IgG conjugated to horseradish peroxidase 1:10 000; (Sigma‐Aldrich Cat# A5420, RRID:AB_258242) for 1 h at room temperature, and antigen–antibody complexes were detected using an enhanced chemiluminescence system (Amersham Biosciences UK). Where indicated, western blots were scanned, and the integrated intensity of each band was determined using ImageJ (RRID:SCR_003070). Results were expressed as a ratio to loading control within the same sample.

### 2.9 Statistical Analysis

ELISA data was expressed as either secretion (pg or ng/ml) per mg of cellular protein. Where variances were unequal, data was either log10 transformed (western blot) or expressed as fold stimulation (ELISA) for statistical analysis. Unpaired *t*‐test, analysis of variance (ANOVA) and linear regression were performed using GraphPad Prism (RRID:SCR_002798). Significance was accepted at *p < 0.05.

## 3 Results

### 3.1 Decidual Expression of MMP‐10

**FIGURE 1:**
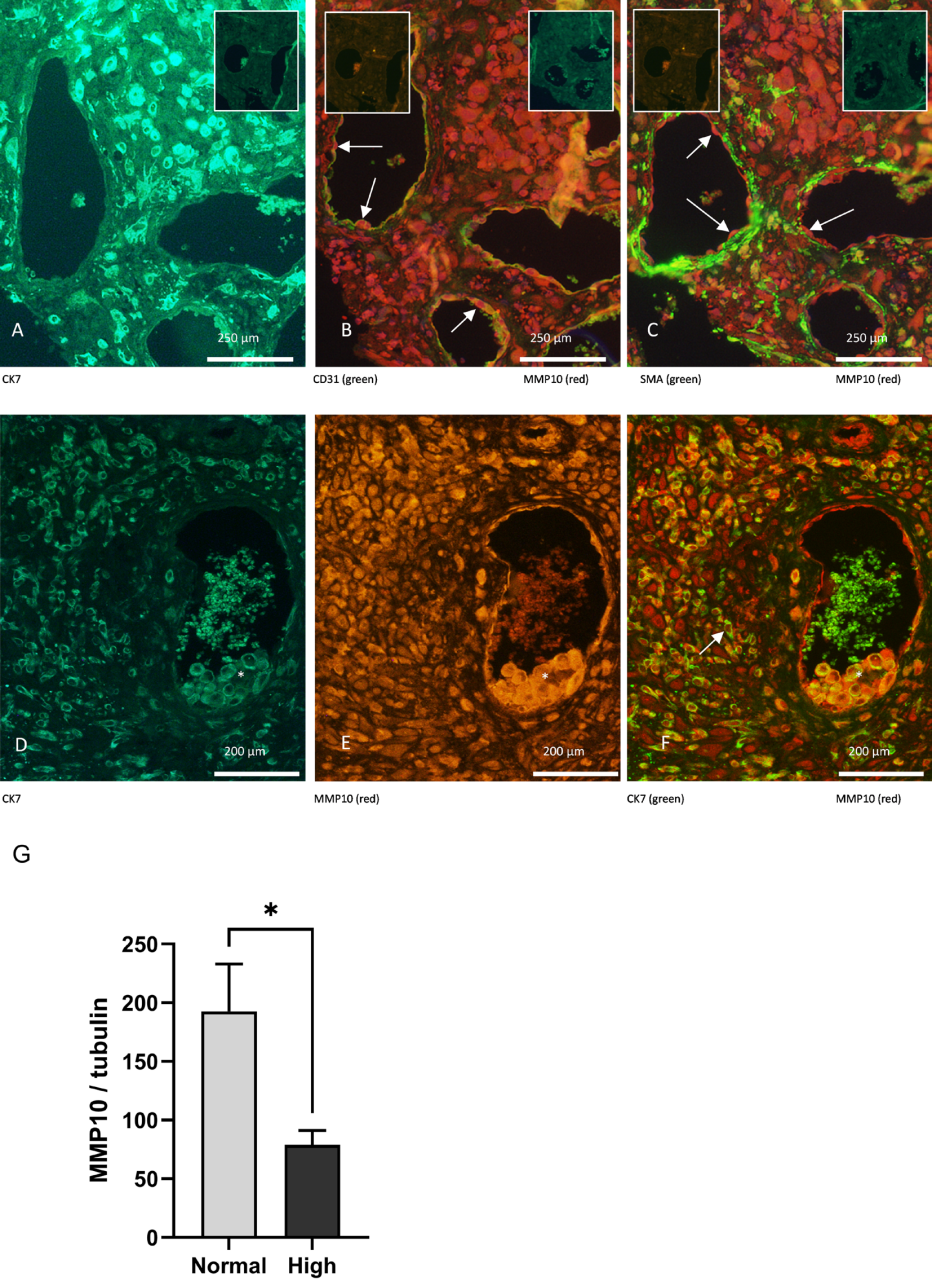
Immunofluorescent staining of first trimester placental bed decidua. (A–C) expression of MMP10 in vascular cells (A) Immunofluorescent staining of CK7 (trophoblasts; green) (B) MMP‐10 (orange/red) and CD31 (endothelial cells; green) and (C) MMP‐10 (orange/red) and smooth muscle actin (VSMC; green). White arrows indicate endothelial cells. The inserts panels show the appropriate non‐immune IgG negative control stained. (D–F) expression of (D) CK7 (E) MMP10 and (F) co‐localisation of CK7 and MMP‐10. The trophoblast plug is highlighted by and asterisk while the arrow indicates a dual‐stained extravillous trophoblast. (G) Expression of MMP10 in 1st trimester decidua from normal pregnancies and those at increased risk of developing PE. Decidual tissue was homogenized in RIPA buffer and the protein separated by gel electrophoresis. Membranes were then probed for MMP10 and tubulin which was used as a loading control. The data are expressed as a ratio of the MMP10 to tubulin and are the mean ± SEM (*n* = 8 per risk group). The level of significance was determined using unpaired *t*‐test where *p < 0.05.

Sections of decidua basalis were stained for MMP‐10 and either smooth muscle actin (VSMC) or CD31 (endothelial cells). Coincident staining was found with CD31, as indicated by the arrows (Figure 1B), there was no co‐localisation of MMP‐10 with α smooth muscle cell actin (Figure 1C). MMP‐10 was also expressed by both interstitial EVT and endovascular trophoblasts that plug and are resident within the lumen of SpA, as determined by coincident staining with CK7 (Figure 1F).

### 3.2 Expression of MMP10 in First Trimester Decidual Tissue From Pregnancies at Increased Risk of Developing Pre‐Eclampsia

Decidual samples isolated from first trimester pregnancies assigned normal or high risk of developing PE were analyzed by western blot. α‐Tubulin was used as a loading control and expression of MMP10/tubulin was calculated. The expression of MMP10 was significantly lower (*p* = 0.02) in samples from pregnancies at increased risk of developing PE compared to the normal risk group (Figure 1G).

### 3.3 Effects of TCM on MMP‐10 Secretion by EC and VSMC

**FIGURE 2:**
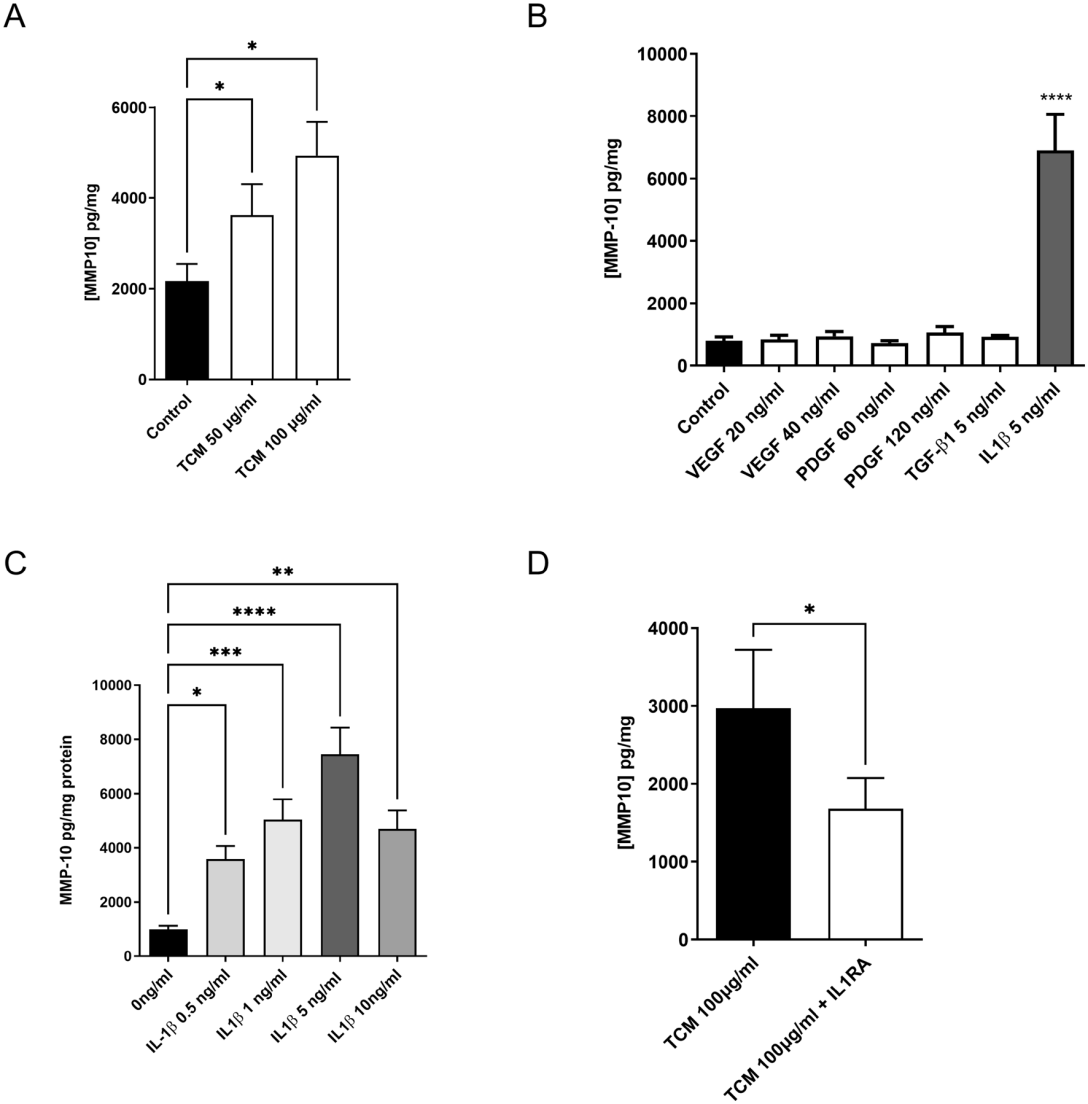
The effect of TCM and IL1β on the secretion of MMP‐10 by EC. (A) EC were stimulated with TCM for 2 h before the medium was removed and the cells were washed extensively with PBS. The cells were then incubated with phenol red free RPMI containing 5% (v/v) FCS for 72 h. (B) EC were serum starved overnight and then stimulated with recombinant growth factors and cytokines for 2 h, washed and incubated as above for 72 h. (C) EC cells were stimulated with increasing concentrations of IL‐1β for 2 h, washed and incubated for 48 h as detailed above. (D) EC cells were either incubated alone or with IL‐1RA for 20 min prior and stimulated with TCM for 2 h, washed and incubated as above for 48 h. At the end of the experiments the concentration of MMP‐10 was determined by ELISA. The results were corrected for total cellular protein and expressed as mean + SEM. Where appropriate the results were analyzed by either a Student‐*t* test, a one‐way ANOVA or a mixed‐effect model using Graph‐pad PRISM. (*p < 0.05, **p < 0.01, ***p < 0.001 ****p < 0.0001).

We have previously reported that *MMP‐10* gene expression was stimulated by TCM using a 3D vascular cell co‐culture system [19]. However, the cellular origin of this expression in this model was not identified. To address this, both EC and VSMC were cultured separately and stimulated with TCM. There was no detectable MMP‐10 secreted by VSMC following stimulation with either concentration of TCM used in this study. However, there was a significant, dose‐dependent increase in secretion by EC found at both concentrations of TCM used in this study (Figure 2A). We also determined that SGHPL‐4 cells, the trophoblast cell line used to produce the TCM, also secreted MMP‐10.

### 3.4 Interleukin 1β but Not TGF‐β, VEGF, or PDGF Stimulates MMP‐10 Secretion by EC

Using a commercially available angiogenesis array, we identified several growth factors and cytokines present in the TCM, including IL‐1β, VEGF, PDGF, and TGF‐β (data not shown). Of the recombinant factors tested, only IL1‐β had a significant effect on MMP‐10 secretion by EC (Figure 2B). We therefore looked at this more closely and were able to demonstrate a dose‐dependent increase in the secretion of MMP‐10 in response to stimulation with IL‐1β with a threshold for stimulation of approximately 0.5 ng/mL. Maximal 4.8‐fold secretion was reached at approximately 5 ng/mL IL‐1β, *p* < 0.0001 (Figure 2C).

### 3.5 Interleukin‐1 Receptor Antagonist Inhibits TCM Stimulated MMP‐10 Secretion

To determine whether IL‐1β present in TCM contributed to MMP‐10 secretion by EC, we pre‐incubated TCM with IL‐1 receptor antagonist (IL‐1RA), an endogenous inhibitor of IL‐1β. This resulted in a significant (*p* < 0.05) drop in the accumulation of MMP‐10 by approximately 40% (Figure 2D).

### 3.6 Intracellular Pathways Involved in TCM and IL‐1β‐Stimulated MMP‐10 Secretion

**FIGURE 3:**
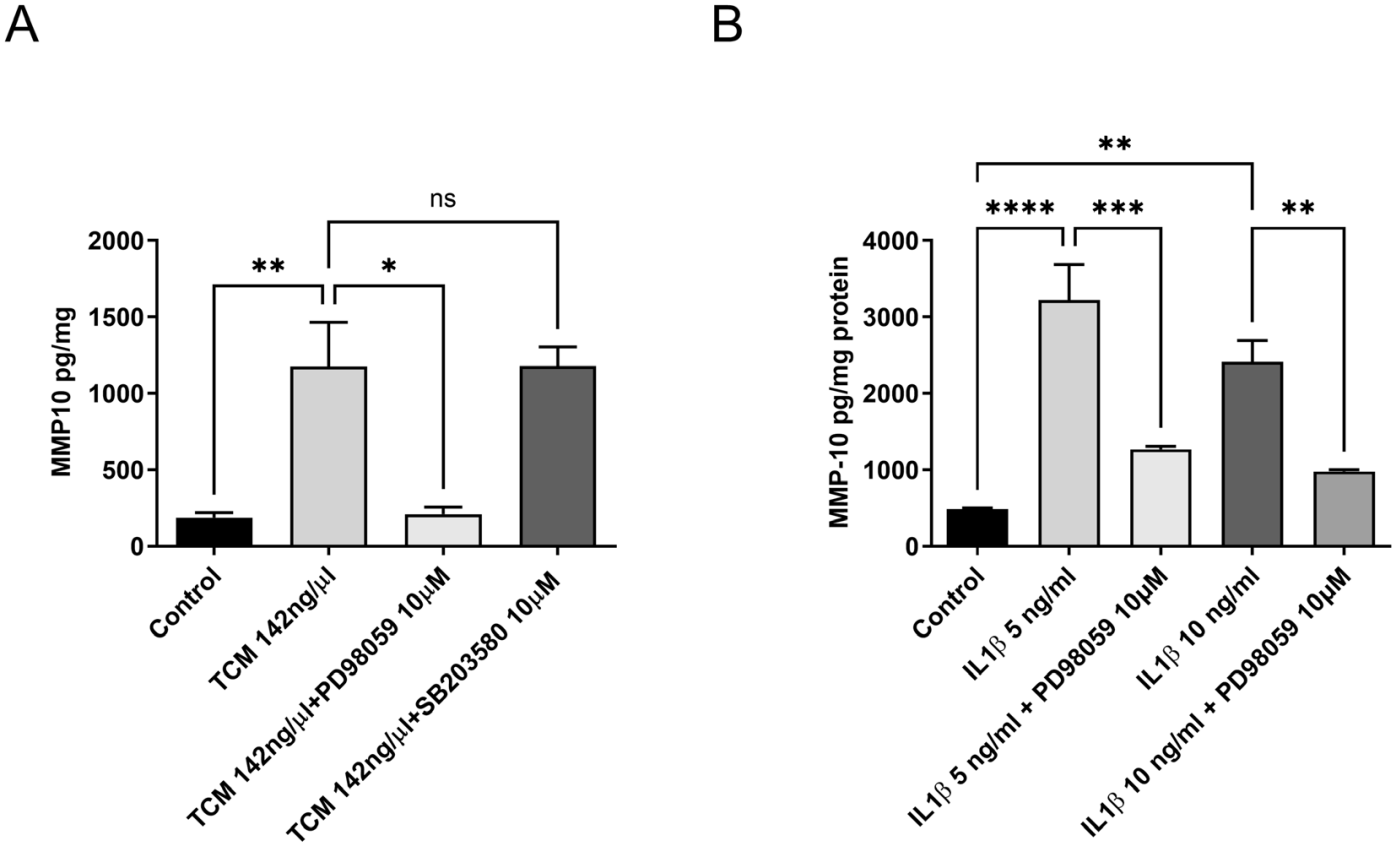
Effect of different pathway inhibitors on TCM and IL‐1β stimulation of EC. (A) EC were incubated with either PD98059, SB203580, or DMSO vehicle for 20 min prior to the addition of TCM (142 ng/mL) for 2 h before being replace with phenol red free RPMI containing 5% (v/v) FCS. After 48 h the medium was removed and MMP‐10 determined by ELISA. (B) EC cells were incubated in the presence and absence of 10 μM PD98059 or DMSO for 20 min prior to the addition of IL‐1β at 5 and 10 ng/mL for 2 h before being replace with phenol red free RPMI containing 5% (v/v) FCS. After 48 h the medium was removed and MMP‐10 determined by ELISA. The results were corrected for protein and expressed as the mean + SEM. (*n* = 3, ns is not significant, *p < 0.05, **p < 0.01, ***p < 0.001 ****p < 0.0001).

**FIGURE 4:**
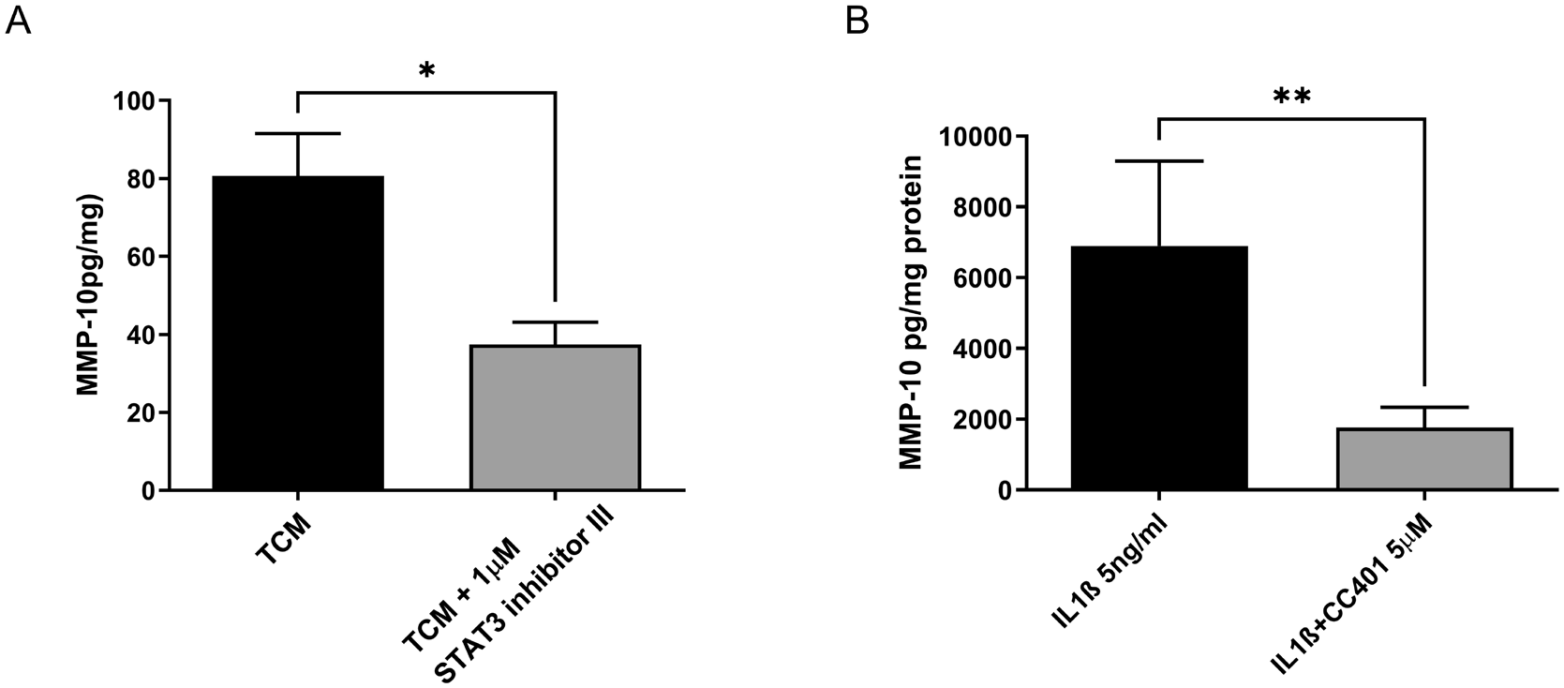
Inhibition of JNK and STAT3 inhibits IL‐1β and TCM‐stimulated MMP‐10 secretion by EC. (A) EC cells were incubated in the presence and absence of STAT3 inhibitor III for 20 min prior to the addition of 50 ng/mL TCM for 2 h. (B) EC cells were incubated in the presence and absence of JNK inhibitor, CC401 or DMSO for 20 min prior to the addition of IL‐1β at 5 for 2 h. After 2 h the medium was removed and replaced with phenol red free RPMI containing 5% (v/v) FCS. After 48 h the medium was removed and MMP‐10 determined by ELISA. This medium was removed and replaced with phenol red free RPMI containing 5% (v/v) FCS. After 48 h the medium was removed and MMP‐10 determined by ELISA. The results were corrected for total cellular protein and expressed as the mean + SEM (A *n* = 5, B *n* = 3 independent experiments, *p < 0.05 and **p < 0.01).

In other cell types, activation of the IL‐1β receptor leads to the activation of different kinases including p42/44‐MAPK, p38‐MAPK, and JNK. The aim of this study was therefore to determine which if—any of—these pathway intermediates may be involved in the regulation of IL‐1β‐stimulated MMP‐10 secretion by EC. Inhibition of p42/44‐MAPK with the MEK inhibitor, PD98059, had a significant inhibitory effect on the secretion of MMP‐10 in response to both TCM and IL‐1β (Figure 3). Inhibition of p38‐MAPK with SB203580 had no effect on either TCM or IL‐1β‐stimulated MMP‐10 secretion (Figure 3A). Inhibition of STAT‐3 had a significant effect on the secretion of TCM‐stimulated MMP‐10 (Figure 4A) but did not significantly affect IL‐1β induced expression. Inhibition of JNK with CC401 significantly inhibited IL‐1β‐stimulated secretion of MMP10 (Figure 4B).

### 3.7 IL‐1β Stimulated sHB‐EGF Secretion

**FIGURE 5:**
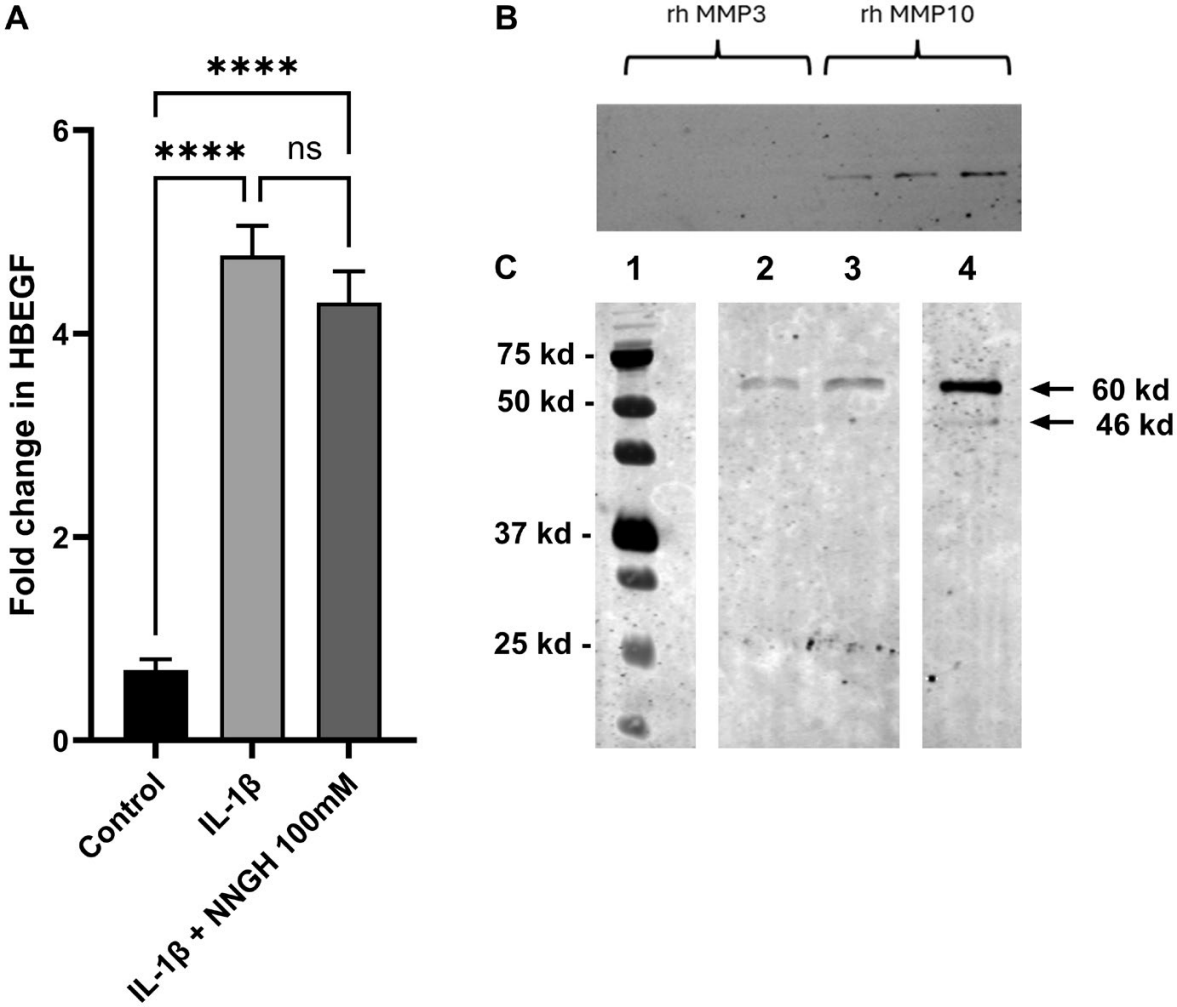
The effect of IL‐1β release of HBEGF and secretion of MMP10 by endothelial cells in culture. (A) EC (0.5 × 10^6^ per dish) were incubated in 0% (v/v) FCS media for 72 h with 5 ng/mL IL‐1β with or without 100 mM NNGH. The concentration of HBEGF was determined by ELISA and corrected for cellular protein. The results are expressed as fold change in HBEGF and is the mean ± SEM of *n* = 4 independent experiments. The level of significance was determined using one‐way ANOVA with Sidak's multiple comparisons test where ns, not significant, ****p < 0.0005. (B) rh‐MMP3 and rh‐MMP10 (2.5‐10 ng/well) was subjected to PAGE, transferred and probed with the R&D systems antibody to determine specificity. (C) EC (0.75 × 10^6^ cells per dish) were cultured for 48 and the medium then removed and concentrated as detailed in the methods. Lane 1 is the molecular size ladder lane 2 control cells, lane 3 cells stimulated with 10 ng/mL IL1β and lane 4 is recombinant MMP10. MMP10 was detected by chemiluminescence and is representative of *n* = 3 independent experiments. The molecular weight of bands obtained in this experiment were calculated using Image Lab v6.1 by BioRad.

It has previously been reported that MMP‐10 can cleave the membrane‐bound form of HB‐EGF to release sHB‐EGF, while we have found that TCM stimulates both MMP‐10 and HB‐EGF mRNA expression. We therefore tested the hypothesis that IL‐1β may stimulate an increase in sHB‐EGF release by EC and that this increase could be prevented by inhibiting MMP‐10 activity. Simulating EC with IL‐1β significantly stimulated the accumulation of HB‐EGF; however, inhibition of MMPs using the broad‐spectrum inhibitor NNGH had no significant effect on the accumulation of HB‐EGF in the medium (Figure 5A).

### 3.8 MMP‐10 Secreted in Response to IL‐1β Secretion Is Largely Inactive

To address possible concerns regarding the cross‐reaction of the MMP‐10 antibody used in this study with MMP‐3, we ran a gel with both rhMMP‐3 (Peprotech Cat# 420–03) and rhMMP‐10 (maximum concentration 10 ng/well) and probed using anti‐MMP‐10 (R and D Systems Cat# MAB9101, RRID:AB_2144565). There was no significant cross‐reaction, as demonstrated in Figure 5B. Using this antibody, we determined that the MMP‐10 was secreted in an inactive pro‐form with a molecular weight of approximately 60 kD. It can then undergo proteolytic cleavage to produce an active form of approximately 46 kD. To determine whether EC can both secrete and activate pro‐MMP‐10, EC were stimulated with IL‐1β. Although we were able to detect the 60 kD pro‐form of MMP‐10 by western blot analysis (Figure 5C), there was no detectable presence of the smaller 46 kD active form.

## 4 Discussion

SpA transformation requires the co‐ordination of several biological processes that result in significant changes to the structure and cellular composition of the vessel wall. Key to many of these events are members of the proteolytic family of enzymes that include MMP‐2, ‐9, and ‐12 [10, 15, 16]. In this study, we examined the novel involvement of MMP‐10. MMP‐10 is a broad‐spectrum matrix metalloproteinase that may be involved in vascular remodeling through its ability to degrade several important components present in the wall of maternal SpA, including collagen type IV, elastin, fibronectin, and laminin [2, 20]. MMP‐10 also takes part in the enzymatic activation of pro‐MMP‐1, ‐2, and ‐9 [21]. More recently, MMP‐10 activity has been associated with the release of bioactive peptides, including HB‐EGF from the cell surface [22].

In the present study, we found that MMP‐10 was widely expressed by cells at the maternal–fetal interface in the first trimester. Co‐localisation was found with cytokeratin 7‐positive trophoblasts, and MMP‐10 was also expressed by decidual stromal cells. These findings are consistent with previous studies reporting the expression of MMP‐10 mRNA in decidual stromal cells and cytotrophoblasts [30, 31, 32]. We also observed co‐localisation of MMP‐10 with CD31/PECAM‐positive cells that line the lumen of the SpA, indicative of EC. However, there was no expression associated with the vascular smooth muscle cell actin, a result supported by our failure to detect the secretion of MMP‐10 by VSMC in culture.

MMP‐10 is a lesser studied proteolytic enzyme but was the only MMP mRNA to be significantly up‐regulated in our in vitro 3D‐model of trophoblast‐induced vascular transformation [19]. Although this may be the first report of the role of MMP‐10 in the transformation of SpA, MMP‐10 has been reported to influence integrity in other vascular beds and is associated with both aortic aneurysms and the pathogenesis of vascular complications associated with type 1 diabetes [33, 34, 35, 36, 37]. In vitro, MMP‐10 plays an important role in cell proliferation, migration, differentiation, and angiogenesis, as well as stimulating the collapse of capillary networks through the activation of MMP‐1 [38]. In vivo *and* in vitro *Mmp‐10* null mice models have been used to study many of the roles of MMP‐10; however, they have not reported reproductive difficulties. On close examination, this is perhaps not surprising as, although there are similarities between placentation in humans and some laboratory animals, there is no natural animal model for either fetal growth restriction or PE [39]. Where the effects of placental insufficiency have led to symptoms such as FGR or PE, spiral artery remodeling remained unaffected [40, 41].

Trophoblasts secrete an array of growth factors and cytokines that could stimulate MMP‐10 secretion by EC, including VEGF, PDGF, TGF‐β and IL1‐β but only the latter significantly stimulated the secretion of MMP‐10 in this study. Using the naturally occurring IL‐1 receptor antagonist, we were able to confirm that IL‐1β was, in part, responsible for the TCM‐stimulated MMP‐10 secretion observed in this study. However, in vivo, trophoblasts are not the only source of IL‐1β as both decidual macrophages and EC express and secrete IL‐1β [19, 42]. This, therefore, raises the possibility that the secretion of MMP‐10 could be under both paracrine and autocrine regulation, independent of trophoblast invasion.

IL‐1β is an important regulator of endothelial cell function in both physiological and pathophysiological circumstances as reviewed elsewhere [43]. IL‐1β acts through the IL‐R to stimulate several intracellular signaling intermediates including NFκB, JNK, p38‐ and p42/44‐MAPK, resulting in changes in gene expression. Using pharmacological inhibitors to common intracellular pathways, we were able to implicate the activation of both p42/44‐MAPK and JNK but not p38‐MAPK in the secretion of MMP‐10 by EC in response to IL1‐β and TCM. These results are in line with those of others that document the activation of multiple kinases in response to IL1‐β [44]. We were also able to implicate STAT3 activation in response to TCM but not IL1‐β, suggesting, as might be predicted, that factors present in TCM other than IL1‐β have a role to play in the secretion of MMP10.

Expression of MMP‐10 by human endometrial EC has been reported [45] and the expression was increased in EC isolated from endometrial cancer compared to controls [46]. MMP‐10 can influence remodeling directly or indirectly through the proteolytic activation of MMP‐1, ‐8, and ‐9. In vitro, MMP‐10 stimulates capillary network collapse through the activation of MMP‐1 [38]. In vivo, MMP‐10 is associated with disease severity and mortality in patients with peripheral arterial disease [47]. Expression of MMP‐1 at the fetal maternal interface has been implicated with trophoblast invasion while aberrant expression both in vivo and in vitro has been associated with PE [48, 49, 50]. There are also reports that MMP‐10 activates matrix‐remodeling programs in macrophages [51], suggesting a possible interaction between SpA EC and the macrophages that cluster around them early in the first trimester [52, 53]. It is also possible that MMP‐10 acts on components of the ECM such as elastin, which may lead to the production of biologically active peptides that stimulate trophoblast migration and invasion, as reported recently [16].

The physiological role played by MMP‐10 at the maternal–fetal interface is still unknown. In this study, we examined the possibility that MMP‐10 increased the release of sHB‐EGF from the EC surface. We were able to demonstrate that IL‐1β stimulated the secretion or release of both MMP‐10 and HB‐EGF by EC; however, the broad‐spectrum MMP inhibitor NNGH [54] had no effect on the concentration of sHB‐EGF present in the media. We therefore looked at other possible biological endpoints, such as invasion, and found no changes. Like many other MMPs, MMP‐10 is secreted in an inactive pro form. The lack of a response in many of our assays led us to question whether the MMP‐10 released by EC in response to stimulation was active. To address this, we performed western blot analysis to look at the expression of MMP‐10. The pro‐form of MMP‐10 has a reported molecular weight of approximately 56 kDa, while the cleaved, active form is approximately 47 kDa [21]. Western blot analysis showed the presence of inactive pro‐MMP‐10, suggesting that EC in this model lacks the ability to proteolytically cleave pro‐MMP‐10.

In conclusion, we have found that MMP‐10 is widely expressed at the maternal–fetal interface, with the most notable exception being the vascular smooth muscle cells of the SpA. In first trimester decidual tissue from pregnancies at increased risk of developing PE, the expression of MMP‐10 is significantly reduced compared to those from a normal pregnancy, suggesting a possible role for MMP‐10 in the normal remodeling process. Using IL1Ra, we could demonstrate that one of the factors secreted by trophoblasts which stimulates MMP‐10 secretion was IL‐1β. Finally, we demonstrated that activation of both p42/44MAPK and JNK was involved in this response. These results are summarized in the graphical abstract. As MMP‐10 has numerous biological activities including the breakdown of ECM, we suggest that it may play a role in SpA remodeling at the maternal–fetal interface, and this warrants further study, perhaps using a multicellular co‐culture system.

## Author Contributions

The study was conceived and planned by G.S.W. and J.E.C. Most of the experimentation was performed by H.T., Z.T., M.A., C.L., N.L., and S.A. The recruitment of the patients for the study was overseen by A.F. The first draft of the paper was written by G.S.W. All authors contributed to the editing of the manuscript.

## Disclosure

The authors have nothing to report.

## Conflicts of Interest

The authors declare no conflicts of interest.

## Data Availability

The datasets generated during and/or analyzed during the current study are available from the corresponding author upon reasonable request.
